# The Long Noncoding RNA Gm9866/Nuclear Factor-*κ*B Axis Promotes Macrophage Polarization

**DOI:** 10.1155/2023/9991916

**Published:** 2023-01-29

**Authors:** Xiaomin Liao, Xianxian Ruan, Xiubing Chen, Biaolin Zheng, Xianbin Wu, Zhejun Deng, Zhe Yang, Feng Wang, Shanyu Qin, Haixing Jiang

**Affiliations:** ^1^Department of Gastroenterology, The First Affiliated Hospital of Guangxi Medical University, Nanning, Guangxi 530021, China; ^2^Department of Gastroenterology, The Third Affiliated Hospital of Guangxi Medical University, Nanning, Guangxi 530000, China

## Abstract

Macrophages are a type of immune cells with high levels of plasticity and heterogeneity. They can polarize into M1 or M2 functional phenotypes. These two phenotypes exhibit a dynamic balance during polarization-related diseases and play opposing roles. Long noncoding RNAs (lncRNAs) play an important role in biological processes such as cell proliferation, death, and differentiation; however, how long noncoding RNAs affect the cellular functionality of macrophages remains to be studied. Long noncoding RNA Gm9866 was found to be closely related to macrophage polarization through bioinformatics analysis. In this study, by conducting real-time polymerase chain reaction analysis, it was observed that long noncoding RNA Gm9866 expression significantly increased after treatment with interleukin-4 but significantly decreased after treatment with lipopolysaccharide. Fluorescence *in situ* hybridization revealed that long noncoding RNA Gm9866 was expressed mainly in the nucleus. Real-time polymerase chain reaction analysis showed that overexpression of long noncoding RNA Gm9866 in RAW264.7 cells further promoted the expression of M2 markers MRC1 (macrophage mannose receptor 1) and MRC2 (macrophage mannose receptor 2). Western blotting analysis demonstrated inhibition of nuclear factor-*κ*B (NF-*κ*B) expression. EdU (5-ethynyl-2′-deoxyuridine) and TUNEL (TdT-mediated dUTP nick-end labeling) staining assays revealed that overexpression of long noncoding RNA Gm9866 promoted cell proliferation and inhibited apoptosis. These findings thus indicated that long noncoding RNA Gm9866 promoted macrophage polarization and inhibited the nuclear factor-*κ*B signaling pathway. Thus, long noncoding RNA Gm9866 may serve as a potential diagnostic and therapeutic target for polarization-related diseases such as infectious diseases, inflammatory diseases, liver fibrosis, and tumors.

## 1. Introduction

Macrophages are immune cells that not only phagocytize virus particles and remove pathogens but also present antigens and secrete cytokines; they also participate in various inflammatory reactions, immune tolerance, and damage to hepatocytes. Macrophages play a dual role in immune response. They have high plasticity and can polarize into different phenotypes and play different functions under the action of different immune immunological conditions [1, 2]. Lipopolysaccharides (LPS) and interferon-*γ* (IFN-*γ*) can promote the differentiation of macrophages into classically activated M1-type; M1 macrophages produce inflammatory factors in various chronic inflammatory diseases such as atherosclerosis [3], inflammatory bowel disease [4], and insulin resistance-related obesity [5] and promote the body's inflammatory response. Interleukin-4 (IL-4) and interleukin-13 (IL-13) induce macrophages to selectively activate the M2 phenotype. After polarization, M2-type macrophages produce anti-inflammatory factors such as interleukin-10 (IL-10) and transforming growth factor *β* (TGF-*β*) to reduce inflammatory response, oxidative stress, and inflammatory injury and promote tissue repair [1, 2]. Therefore, macrophages have both proinflammatory and anti-inflammatory effects, which depend on their specific phenotypes in the disease. Macrophage polarization is also related to tumor. Luo et al. found that long noncoding RNA Ma301 associated with macrophage polarization regulated the Akt/ERK1 (extracellular signal-regulated kinase 1) pathway by interacting with caprin-1, which inhibited the proliferation, migration, epithelial-mesenchymal transition, and lung metastasis of liver hepatocellular cells [6]. Intervention against macrophages with specific phenotypes could serve as one of the potential targets for treating diseases. The long noncoding RNAs (lncRNAs) are a form of noncoding RNA with a length greater than 200 nucleotides (nt). They can directly participate in the epigenetic, transcriptional, and posttranscriptional regulation of mRNA and can also regulate target genes by competitive inhibition of miRNA by sponge action [7–9]. Several long noncoding RNAs have been found to be related to the pathogenesis of macrophage polarization. For example, Pi et al. found that long noncoding RNA XIST targeted interleukin-33/micro-RNA-19b to promote the proliferation and migration of skin fibroblasts; this long noncoding RNAs also promoted the formation of fibroblast extracellular matrix by facilitating the transformation of macrophages to M2 phenotype [10]. In another study, Yang et al. showed that in a subcutaneous tumor model, extracellular vesicles (EVS) derived from bone marrow mesenchymal stem cells (BMSCs) carrying long noncoding RNA NEAT1 could promote the progression of melanoma by inducing M2 polarization of macrophages [11]. Wang et al. reported that exosomal long noncoding RNA HMMR-AS1 mediated macrophage polarization through micro-RNA-147a/ARID3A (AT-rich interaction domain 3A) axis under hypoxia, which affected the progression of hepatocellular carcinoma [12]. However, in recent years, very few studies have been conducted on the relationship between long noncoding RNAs and macrophage polarization, and more macrophage-related long noncoding RNAs need to be studied. In the present study, we analyzed the microarray datasets of two mouse macrophages (GSE107952 and GSE107979) [13] and screened several long noncoding RNAs that were closely related to the polarization process of macrophages. Of these, long noncoding RNA Gm9866 showed dramatic changes in expression during the polarization process of macrophages. However, whether long noncoding RNA Gm9866 plays an important role in macrophage polarization and its specific mechanism remain unknown. The present study reports the relationship between long noncoding RNA Gm9866 and macrophage polarization for the first time.

It is hypothesized that long noncoding RNA Gm9866 promotes cell proliferation and M2 polarization and inhibits cell apoptosis in macrophages via the nuclear factor-*κ*B (NF-*κ*B) pathway. Thus, the present study is aimed at identifying the roles of long noncoding RNA Gm9866 in cell proliferation, polarization, and apoptosis and to further explore its potential mechanisms during M2 polarization of RAW 264.7 cells. These experimental results revealed a novel role of long noncoding RNA Gm9866 in the polarization of macrophages, which may contribute to the development of targeted therapeutic strategies for macrophage polarization-related diseases such as atherosclerosis, inflammatory bowel disease, insulin resistance-related obesity, and tumors.

## 2. Material and Methods

### 2.1. Identification of Differentially Expressed Long Noncoding RNAs between M1 and M2 Macrophages

To screen for the functional long noncoding RNAs involved in macrophages M2 polarization, we selected two microarray datasets (GSE107952 and GSE107979) [13] from Gene Expression Omnibus database. The GSE107952 dataset included RAW264.7 cellular model of interleukin-13-driven macrophage M2 polarization (four groups). The GSE107979 included bone marrow-derived macrophages (BMDMs) treated with interleukin-13 (two groups). The raw data of the datasets were preprocessing via background adjustment, quantile normalization, final summarization, and log2 transformation. Then, “limma” (linear models for microarray data) package in R language was used to screen differentially expressed long noncoding RNAs between M1 and M2 macrophage. The differentially expressed long noncoding RNAs with a *p* value less than 0.05 was considered significant. Next, the common differentially expressed long noncoding RNAs from the two datasets were identified by overlapping them [14].

### 2.2. Cell Culture

RAW 264.7 cells were purchased from the American Type Culture Collection (Manassas, VA, USA). The cells were cultured in Dulbecco's modified Eagle medium (Gibco, Gaithersburg, MD, USA) supplemented with 10% heat-inactivated fetal bovine serum (FBS; BI; VivaCell, Shanghai, China), 1% penicillin/streptomycin, 1% sodium pyruvate, and 3.7 g/L sodium bicarbonate at 37°C under 5%CO_2_ [15].

### 2.3. Induction of Macrophage Differentiation

RAW 264.7 cells were serum-starved for 18 h and then divided into three groups: (1) control; (2) LPS (lipopolysaccharide; Sigma-Aldrich, St. Louis, Missouri, USA) at concentrations of 100 ng/mL, 1 *μ*g/mL, 2 *μ*g/mL, 4 *μ*g/mL, and 8 *μ*g/mL; and (3) IL-4 (interleukin-4; PeproTech, Rocky Hill, NJ, USA) at concentrations of 1 ng/mL, 2 ng/mL, 3 ng/mL, 4 ng/mL, and 5 ng/mL. Each treatment was for an additional 24 h [15].

### 2.4. Fluorescence *In Situ* Hybridization (FISH)

Subcellular localization of long noncoding RNA Gm9866 was detected by fluorescence *in situ* hybridization assays (RiboBio, Guangzhou, China). The cells were fixed with 4% paraformaldehyde (Servicebio, Wuhan, China), prehybridized, and then immersed in hybridization solution containing the long noncoding RNA Gm9866 probe marked with cyanine 3 (Cy3) and incubated overnight at 37°C. Then, the cells were stained with 4′,6-diamidino-2-phenylindole (RiboBio, Guangzhou, China). Images were then acquired by laser scanning confocal microscopy (TCS SP8, LEICA, Germany) [16].

### 2.5. Cell Transfection

The long noncoding RNA Gm9866 was overexpressed in RAW 264.7 cells by transfecting the cells with a long noncoding RNAGm9866-overpressing plasmid (Genechem, Shanghai, China) with Advanced DNA RNA Transfection Reagent™ (Zeta Life, Menlo Park, CA, USA) in accordance with the manufacturer's protocol. In brief, RAW264.7 cells were placed on the surface of culture plates 1 day in advance and allowed to grow to 60−80% confluency. Then, the plasmid was directly mixed with transfection regent (1 : 1) and mixed by using a pipette (10–15 times). Following incubation at room temperature for 15 min, the mixture was added to the cell culture plates, mixed gently, and incubated in a CO_2_ incubator for 24 h [17].

### 2.6. RNA Extraction and Real-Time Polymerase Chain Reaction (RT-PCR)

The relative gene expression data were analyzed using a previously reported method [15]. Total RNA was isolated from Raw 246.7 cells by homogenizing liver tissues with a NucleoZOL isolation kit (Macherey-Nagel, Düren, Germany) in accordance with the manufacturer's protocol. Real-time polymerase chain reaction assays were performed using Prime ScriptTM RT Master Mix (Perfect Real Time) reagent kits (Takara Bio, Shiga, Japan) along with a FastStart Universal SYBR Green Master (ROX) kit (Roche, Mannheim, Germany), in accordance with the manufacturer's instructions. The following primers were used: mouse long noncoding RNA Gm9866, 5′-TGG TTG CTT GTT GAT GCC TCC TG-3′ (forward) and 5′-GTG CCT TCT GTG ACC CTG TGT G-3′ (reverse); mouse GAPDH, 5′-GGT TGT CTC CTG CGA CTT CA-3′ (forward) and 5′-TGG TCC AGG GTT TCT TTA CTC C-3′ (reverse); mouse MRC2 (macrophage mannose receptor 2), 5′-ATG GCA ACT GGA GGC AAT ATG AG-3′ (forward) and 5′-GGC TGC AGG TCA GCA GGT TTA-3′ (reverse); and mouse MRC1 (macrophage mannose receptor 1), 5′-AGC TTC ATC TTC GGG CCT TTG-3′ (forward) and 5′-GGT GAC CAC TCC TGC TGC TTA G-3′ (reverse). The real-time polymerase chain reaction conditions were as follows: one cycle of 50°C for 2 min and 95°C for 10 min and 40 cycles of 15 s at 95°C and 1 min at 60°C. The target gene mRNA expression was normalized to that of GAPDH. All reactions were performed in triplicate for each sample. At least three independent experiments were carried out for each experimental condition.

### 2.7. Western Blotting

Radioimmunoprecipitation assay (RIPA) buffer (Solarbio, Shanghai, China) was used to lyse cells, and the BCA kit (Beyotime, China) was used to quantify protein levels. *β*-Actin (AbMART, Shanghai, China) was used as a loading control. The primary antibody specific for NF-*κ*B (nuclear factor-*κ*B) was obtained from AbMART (Shanghai, China). Anti-rabbit and anti-mouse second antibodies were obtained from Signalway Antibody (Pearland, TX, USA). An Odyssey two-color infrared laser imaging system (LI-COR Biosciences, Lincoln, NE, USA) was employed to scan the blots. The grey values were quantitatively analyzed using ImageJ software (NIH, USA) [15].

### 2.8. EdU (5-Ethynyl-2′-Deoxyuridine) Assay

Cell proliferation was monitored by EdU (5-ethynyl-2′-deoxyuridine) assays (RiboBio, Guangzhou, China). In brief, cultured RAW264.7 cells were incubated with 50 *μ*M EdU (5-ethynyl-2′-deoxyuridine) solution for 24 h, followed by fixation with 4% paraformaldehyde for 30 min and permeation using 0.5% TritonX-100 for 10 min. The treated cells were stained with an Apollo fluorescent dyeing solution and Hoechst 33,342 for 30 min and observed with an inverted fluorescence microscope (Nikon, Tokyo, Japan). All experiments were performed in triplicate [18].

### 2.9. Apoptosis Assay

TUNEL (TdT-mediated dUTP nick-end labeling) staining kit (Meilun Biotechnology, Dalian, China) was used to determine apoptosis of RAW264.7 cells. The experimental methods were performed strictly in accordance with the manufacturer's instructions. A fluorescence microscope (Nikon, Tokyo, Japan) was used to observe the features of apoptosis. The cells stained with TUNEL (TdT-mediated dUTP nick-end labeling) were considered apoptotic. The total number of apoptotic cells was calculated using DAPI (2-(4-amidinophenyl)-6-indolecarbamidine dihydrochloride) staining [19].

### 2.10. Statistical Analysis

Data of the three independent experiments are presented using themean ± standarddeviation. SPSS version 25.0 (SPSS, Chicago, IL, USA) and GraphPad Prism (GraphPad Software Inc.) were used for all statistical analyses. Data were compared with the Student's *t*-test and one-way analysis of variance (ANOVA). Probability (*p*) values < 0.05 were considered statistically significant [15].

## 3. Results

The present study demonstrated that long noncoding RNA Gm9866 expression was upregulated during the polarization of macrophages toward the M2 type as noted in the published transcriptome dataset and verified in the *in vitro* experiment with RAW 264.7 cells. We also found that interleukin-4 upregulated but lipopolysaccharide (LPS) downregulated the expression of long noncoding RNA Gm9866 in RAW 264.7 cells. Moreover, the phenotypic markers of M2 macrophages increased significantly after the overexpression of long noncoding RNA Gm9866 in RAW 264.7 cells, which promoted M2 polarization and decreased nuclear factor-*κ*B protein expression. The overexpression of long noncoding RNA Gm9866 also promoted the proliferation of RAW 264.7 cells and inhibited apoptosis. These findings suggested that the upregulation of long noncoding RNA Gm9866 may contribute to the progression of macrophage polarization-related diseases. Finally, laser confocal microscopy showed that long noncoding RNA Gm9866 was expressed in the cytoplasm and nucleus of macrophages and mainly in the nucleus.

### 3.1. Screening of Differentially Expressed Long Noncoding RNAs

**Figure 1:**
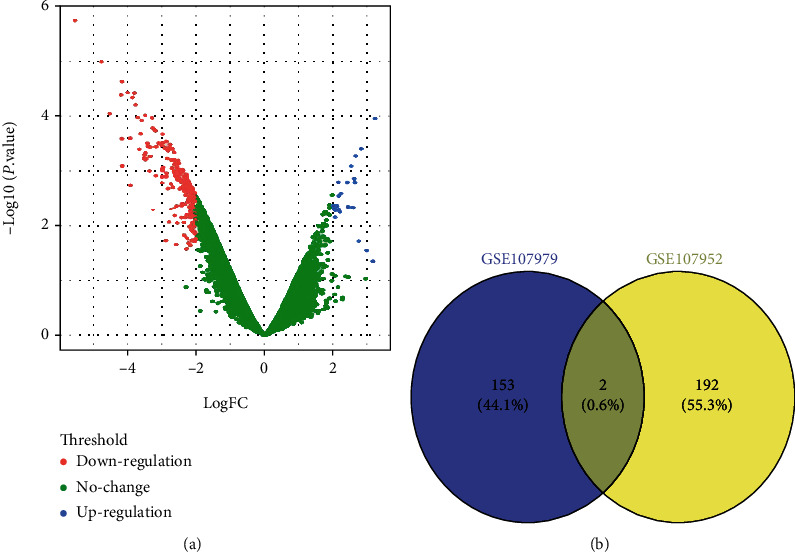
Screening of differentially expressed long noncoding RNAs (lncRNAs). (a) Volcano plot of GSE107979 dataset. (b) Common differentially expressed long noncoding RNAs between the GSE107979 and GSE107952 datasets.

The GSE107979 and GSE107952 datasets [13] were used to screening the differentially expressed long noncoding RNAs between M1 and M2 macrophage. A total of 153 differentially expressed long noncoding RNAs were identified in GSE107979 dataset. After overlapping the results of the of the GSE107979 dataset with those of the GSE107952, two differentially expressed long noncoding RNAs were identified as the common differentially expressed long noncoding RNAs (Gm9866 and AK086120) (Figure 1). Because the role of long noncoding RNA Gm9866 in macrophages was not reported previously, we selected long noncoding RNA Gm9866 for the subsequent experiments.

### 3.2. The Long Noncoding RNA Gm9866/Nuclear Factor-*κ*B Axis Promotes M2 Polarization of Macrophages

**Figure 2:**
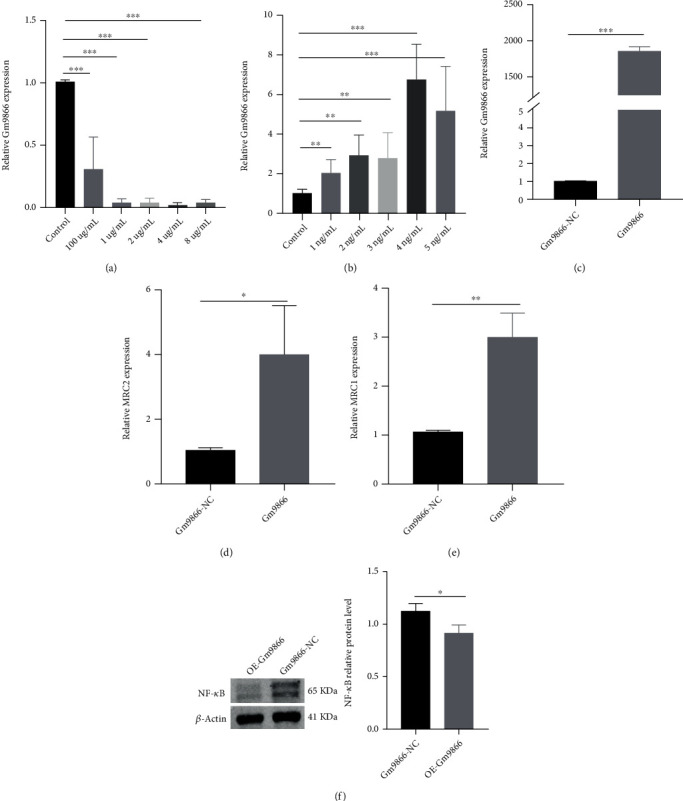
Long noncoding RNA Gm9866 is closely related to macrophage polarization. (a) LPS (lipopolysaccharide) reduced the expression levels of long noncoding RNA Gm9866. (b) IL4 (interleukin-4) increased the expression levels of long noncoding RNA Gm9866. (c) Real-time polymerase chain reaction analysis for the transfection efficiency of long noncoding RNA Gm9866. (d, e) Real-time polymerase chain reaction analysis showed that the mRNA expression of the M2-related genes MRC2 (macrophage mannose receptor 2) and MRC1 (macrophage mannose receptor 1) significantly increased after overexpression of long noncoding RNA Gm9866. (f) NF-*κ*B (nuclear factor-*κ*B) is a target of long noncoding RNA Gm9866. The protein level of NF-*κ*B (nuclear factor-*κ*B) was significantly decreased after overexpression of long noncoding RNA Gm9866. ^∗^*p* < 0.05, ^∗∗^*p* < 0.01, and ^∗∗∗^*p* < 0.001.

To investigate whether long noncoding RNA Gm9866 expression was associated with macrophages polarization, unpolarized macrophages, lipopolysaccharide-induced M1 macrophages, and interleukin-4-induced M2 macrophages were obtained. The expression level of long noncoding RNA Gm9866 was then determined. The expression level of long noncoding RNA Gm9866 was significantly increased after interleukin-4 treatment but was significantly decreased after lipopolysaccharide treatment as compared to that of nonpolarized macrophages (Figures 2(a) and 2(b)); these findings are consistent with the results of bioinformatics analysis [13]. There was no significant correlation between the expression level of long noncoding RNA Gm9866 and the concentration of lipopolysaccharide, while interleukin-4 had different effects on the expression level of long noncoding RNA Gm9866 at different concentrations (no significance for 1 ng/mL vs. 2 ng/mL, 1 ng/mL vs. 3 ng/mL, 2 ng/mL vs. 3 ng/mL, and 3 ng/mL vs. 4 ng/mL; *p* < 0.05 for 1 ng/mL vs. 4 ng/mL, 1 ng/mL vs. 5 ng/mL, 2 ng/mL vs. 4 ng/mL, 2 ng/mL vs. 5 ng/mL, 3 ng/mL vs. 5 ng/mL, and 4 ng/mL vs. 5 ng/mL).

To further assess how long noncoding RNA Gm9866 influences macrophages polarization, a long noncoding RNA Gm9866-overpressing plasmid was transfected into RAW264.7 cells, and the M2 macrophage phenotype markers were then detected. The results showed that the expression of the M2 macrophage markers MRC1 (macrophage mannose receptor 1) and MRC2 (macrophage mannose receptor 2) was significantly increased after the overexpression of long noncoding RNA Gm9866 (Figures 2(c)–2(e)). Previous studies [20, 21] have reported that the nuclear factor-*κ*B family was involved in the process of macrophage polarization. On the basis of the above-mentioned result, it was clear that long noncoding RNA Gm9866 promoted macrophage polarization; hence, we speculated that the nuclear factor-*κ*B pathway was likely to participate in macrophage polarization promoted by long noncoding RNA Gm9866. Therefore, the nuclear factor-*κ*B pathway was selected for further analysis to clarify its specific mechanism. Western blotting assay showed that the expression of nuclear factor-*κ*B protein decreased after overexpression of long noncoding RNA Gm9866, thus suggesting that the nuclear factor-*κ*B pathway plays a role here; in other words, the specific mechanism by which long noncoding RNA Gm9866 promoted macrophage polarization to M2 phenotype could be determined by inhibiting the nuclear factor-*κ*B pathway (Figure 2(f)).

### 3.3. The Subcellular Localization of Long Noncoding RNA Gm9866

**Figure 3:**
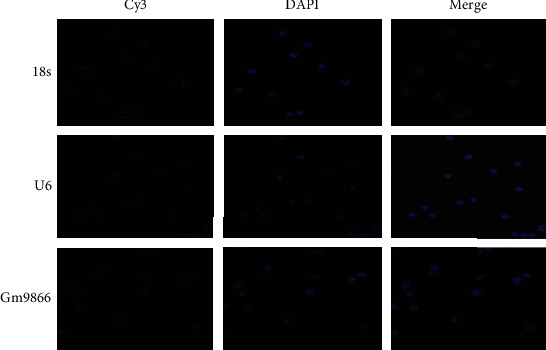
Subcellular localization of long noncoding RNA Gm9866 in RAW24.7 cells. Fluorescence *in situ* hybridization showed that long noncoding RNA Gm9866 was expressed in the nucleus and cytoplasm, but mainly in the nucleus (scale bar, 5 *μ*m).

Fluorescence *in situ* hybridization analysis was conducted to confirm the subcellular localization of long noncoding RNA Gm9866 in RAW24.7 cells. The long noncoding RNA Gm9866 was expressed in the nucleus and cytoplasm, but mainly in the nucleus (Figure 3). This result suggested that long noncoding RNA Gm9866 mainly played a functional role in epigenetic regulation and transcriptional regulation [7–9]; this finding could provide a theoretical foundation for more direct and in-depth research on the mechanism of long noncoding RNA Gm9866.

### 3.4. The Long Noncoding RNA Gm9866 Promoted Macrophage Proliferation

**Figure 4:**
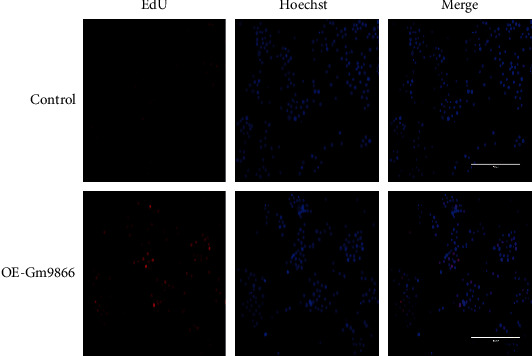
Effect of long noncoding RNA Gm9866 on RAW264.7 cell proliferation. EdU (5-ethynyl-2′-deoxyuridine) showed that long noncoding RNA Gm9866 overexpression promoted the proliferation of RAW264.7 cells (scale bar: 200 *μ*m).

The effect of long noncoding RNA Gm9866 on the proliferation of RAW264.7 cells was detected by EdU (5-ethynyl-2′-deoxyuridine) assay. The results showed that long noncoding RNA Gm9866 upregulation led to enhanced proliferation of RAW264.7 cells (Figure 4). Chen et al. also reported that macrophages polarized to the M2 type exhibited enhanced cell proliferation [22].

### 3.5. The Long Noncoding RNA Gm9866 Inhibited Macrophage Apoptosis

**Figure 5:**
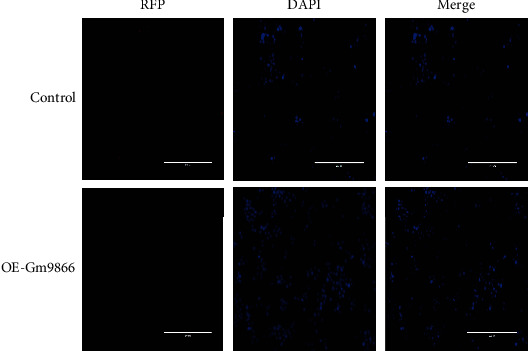
Effect of long noncoding RNA Gm9866 on the apoptosis of RAW264.7 cells. TUNEL (TdT-mediated dUTP nick-end labeling) staining showed that long noncoding RNA Gm9866 overexpression inhibited the apoptosis of RAW264.7 cells (scale bar: 200 *μ*m).

TUNEL (TdT-mediated dUTP nick-end labeling) assay was performed to determine the effect of long noncoding RNA Gm9866 on the apoptosis of RAW264.7 cells. The results showed that long noncoding RNA Gm9866 upregulation suppressed the apoptosis of RAW264.7 cells (Figure 5).

## 4. Discussion

Macrophages are immune cells that play the role of “scavengers” and participate in various cellular and molecular immune pathways [23]. Macrophages also participate in the occurrence and development of many diseases such as sepsis [24], abdominal aortic aneurysm [25], endometriosis [26], brain injury [27], oral squamous cell carcinoma [28], liver fibrosis [15], liver cancer [29], and pancreatic cancer [30]. In recent years, long noncoding RNAs (lncRNAs) have become key molecules in the study of many diseases, and several studies on long noncoding RNAs related to the pathogenesis of macrophage polarization have been reported. These studies have provided new ideas for the clinical treatment of macrophage polarization-related diseases. Previous studies have shown the effects of macrophage-associated long noncoding RNAs on sepsis-induced liver injury; HCC (hepatocellular carcinoma cell) progression, invasion, and metastasis; and immunosuppression [6, 31–35]. As mentioned earlier, macrophages can differentiate into M1 and M2 phenotypes, and these phenotypes play different roles in different diseases. A variety of long noncoding RNAs associated with different polarization states of macrophages have been reported. The expression of long noncoding RNA COX-2 in M1 macrophages was found to be higher than that in nonpolarized macrophages and M2 macrophages [29]. Moreover, long noncoding RNA COX-2 siRNA reduced the ability of M1 macrophages to inhibit HCC (hepatocellular carcinoma cell) cell proliferation, invasion, and migration, EMT (epithelial-mesenchymal transition), angiogenesis, and apoptosis and enhanced the ability of M2 macrophages to promote the proliferation and growth of HCC (hepatocellular carcinoma cell) cells and inhibit apoptosis [29]. Chen et al. conducted a series of cell experiments and reported that knockout of long noncoding RNA PCAT6 inhibited M2 polarization of macrophages and subsequently inhibited the growth of non-small-cell lung cancer cells [36]. Exosomes derived from renal cell carcinoma cells were reported to promote macrophage polarization, cytokine release, phagocytosis, angiogenesis, and tumor development. Overexpression of long noncoding RNA ARSR induced phenotypic and functional changes of macrophages *in vitro* and promoted tumor growth *in vivo*, while knockout of long noncoding RNA ARSR impaired the exosome-mediated polarization of macrophages [37]. Luo et al. established an *in vitro* cell model and an *in vivo* mouse model of *Mycobacterium tuberculosis* infection and conducted negative pressure treatment. They found that negative pressure treatment after *M. tuberculosis* infection promoted macrophage polarization to proinflammatory M1 phenotype by regulating the long noncoding RNA XIST/micro-RNA-125b-5p/A20/nuclear factor-*κ*B axis [38]. Most transcripts of the human genome sequence are composed of noncoding RNAs, one of which is long noncoding RNA [39]. Although several long noncoding RNAs related to macrophages have been reported in recent years, many long noncoding RNAs related to macrophages are yet to be explored. Therefore, in the present study, we analyzed the two microarray datasets of two mouse macrophages (GSE107952 and GSE107979) [13] and found that the expression levels of long noncoding RNA Gm9866 changed dynamically during macrophage polarization. Long noncoding RNA Gm9866 is located at A2 on chromosome 12, which has four exons and three transcripts. Hi-C chromosome conformation data from the mouse brain show that the long noncoding RNA Gm9866 locus is spatially close to that of Sox11 (SRY-related HMG-box gene 11), indicating that there may be a regulatory relationship between these two loci in the nervous system [40]. Presently, there are few studies on the biological role of long noncoding RNA Gm9866. Recently, experiments on a mouse cardiac hypertrophy model showed that long noncoding RNA Gm9866 was highly expressed in hypertrophic myocardium. Long noncoding RNA Gm9866 formed a module with six other long noncoding RNAs, participated in the construction of a long noncoding RNA gene network for a cardiac hypertrophy, and was shown to mediate the occurrence of cardiac hypertrophy [41]. Another study found that long noncoding RNA Gm9866 was involved in the development of the mouse dorsal root ganglion. Knockout of long noncoding RNA Gm9866 has been shown to reduce the expression levels of Sox11 (SRY-related HMG-box gene 11) in nerve cells and delay the recovery process [42]. These studies suggest that long noncoding RNA Gm9866 is involved in the development and injury of some cells. However, there are no previous reports regarding to the role of long noncoding RNA Gm9866 in macrophage polarization. Therefore, the present study was conducted to explore the relationship between long noncoding RNA Gm9866 and macrophage polarization and the underlying mechanism. In an *in vitro* experiment, lipopolysaccharide and interleukin-4 were used to induce cell polarization. The results showed that the expression levels of long noncoding RNA Gm9866 decreased when macrophages were polarized to M1-type and increased when macrophages were polarized to M2-type; this finding was in line with the screening results of the bioinformatics analysis. Cell transfection experiments were also conducted. After transfection of long noncoding RNA Gm9866-overexpressing plasmids, the levels of the M2-specific phenotypic markers MRC1 (macrophage mannose receptor 1) and MRC2 (macrophage mannose receptor 2) were increased, thus suggesting that macrophages are polarized to M2 phenotype, which may play an anti-inflammatory role. Subsequently, a FISH (fluorescence in situ hybridization) analysis was conducted to locate long noncoding RNA Gm9866 in RAW264.7 cells. Long noncoding RNA Gm9866 was found to be expressed in the nucleus and cytoplasm, but mainly in the nucleus. This finding suggested that long noncoding RNA Gm9866 mainly played a functional role in epigenetic regulation and transcriptional regulation. These results laid a foundation for more direct and in-depth research on the molecular mechanism of long noncoding RNA Gm9866. Some previous studies [20, 21, 38, 43–45] have reported that the nuclear factor-*κ*B family is involved in macrophage polarization. We therefore speculated that long noncoding RNA Gm9866 may act through the nuclear factor-*κ*B signaling pathway. Western blotting assay was then performed to detect the protein levels of nuclear factor-*Κ*B. The results showed that the nuclear factor-*κ*B protein level decreased after the overexpression of long noncoding RNA Gm9866, which further confirmed the previous speculation. Taken together, the present study suggested that long noncoding RNA Gm9866 promoted M2 macrophage polarization through the nuclear factor-*κ*B signaling pathway. However, the specific mechanism of this phenomenon and the involved binding sites need to be further studied.

The present study had some limitations that should be addressed in future research. First, bone marrow-derived macrophages should be isolated to further improve the verification of long noncoding RNA Gm9866 through the loss of function experiment. Second, the findings of the present study can be strengthened by *in vivo* experiments and research on specific diseases. The biological function of long noncoding RNA Gm9866 can be further verified by combining pathway enhancers or blockers. Finally, bioinformatics analysis can also be used to predict the direct target genes of long noncoding RNA Gm9866 for more in-depth and direct research on the underlying mechanism. Despite these limitations, the present study is the first to explore the relationship between long noncoding RNA Gm9866 and macrophage polarization. These results provided new insights into the molecular mechanism by which long noncoding RNA Gm9866 was involved in macrophage polarization-related diseases.

## 5. Conclusions

In conclusion, the present study revealed the potential mechanism by which long noncoding RNA Gm9866 promoted macrophage proliferation and polarization and inhibited its apoptosis by inhibiting the nuclear factor-*κ*B pathway. Therefore, targeting long noncoding RNA Gm9866 and nuclear factor-*κ*B can serve as a new therapeutic strategy for treating macrophage polarization-related diseases.

## Data Availability

All data generated or analyzed during this study are included in this article. The data is available from the corresponding author on reasonable request.
